# Opposite modulation of functional recovery following contusive spinal cord injury in mice with oligodendrocyte-selective deletions of *Atf4* and *Chop/Ddit3*

**DOI:** 10.1038/s41598-023-36258-2

**Published:** 2023-06-06

**Authors:** Yonglin Gao, George Z. Wei, Michael D. Forston, Benjamin Rood, Emily R. Hodges, Darlene Burke, Kariena Andres, Johnny Morehouse, Christine Armstrong, Charles Glover, Lukasz P. Slomnicki, Jixiang Ding, Julia H. Chariker, Eric C. Rouchka, Sujata Saraswat Ohri, Scott R. Whittemore, Michal Hetman

**Affiliations:** 1 Kentucky Spinal Cord Injury Research Center, University of Louisville School of Medicine, 511 S. Floyd St., MDR616, Louisville, KY 40202 USA; 2 Department of Neurological Surgery, University of Louisville School of Medicine, Louisville, KY 40292 USA; 3 Department of Anatomical Sciences and Neurobiology, University of Louisville School of Medicine, Louisville, KY 40292 USA; 4 Department of Pharmacology and Toxicology, University of Louisville School of Medicine, Louisville, KY 40292 USA; 5 Department of Biochemistry and Molecular Genetics, University of Louisville School of Medicine, Louisville, KY 40292 USA; 6 MD/PhD Program, University of Louisville School of Medicine, Louisville, KY 40292 USA; 7 Department of Oral Immunology and Infectious Diseases, University of Louisville School of Dentistry, Louisville, KY 40292 USA; 8 Kentucky Biomedical Research Infrastructure Network Bioinformatics Core, University of Louisville, Louisville, KY 40292 USA

**Keywords:** Myelin biology and repair, Diseases of the nervous system, Spinal cord diseases, Stress signalling

## Abstract

The integrated stress response (ISR)-activated transcription factors ATF4 and CHOP/DDIT3 may regulate oligodendrocyte (OL) survival, tissue damage and functional impairment/recovery in white matter pathologies, including traumatic spinal cord injury (SCI). Accordingly, in OLs of OL-specific RiboTag mice, *Atf4, Chop/Ddit3* and their downstream target gene transcripts were acutely upregulated at 2, but not 10, days post-contusive T9 SCI coinciding with maximal loss of spinal cord tissue. Unexpectedly, another, OL-specific upregulation of *Atf4/Chop* followed at 42 days post-injury. However, wild type versus OL-specific *Atf4*^*−/−*^ or *Chop*^*−/−*^ mice showed similar white matter sparing and OL loss at the injury epicenter, as well as unaffected hindlimb function recovery as determined by the Basso mouse scale. In contrast, the horizontal ladder test revealed persistent worsening or improvement of fine locomotor control in OL-*Atf4*^*−/−*^ or OL-*Chop*^*−/−*^ mice, respectively. Moreover, chronically, OL-*Atf*^*−/−*^ mice showed decreased walking speed during plantar stepping despite greater compensatory forelimb usage. Therefore, ATF4 supports, while CHOP antagonizes, fine locomotor control during post-SCI recovery. No correlation between those effects and white matter sparing together with chronic activation of the OL ISR suggest that in OLs, ATF4 and CHOP regulate function of spinal cord circuitries that mediate fine locomotor control during post-SCI recovery.

## Introduction

The integrated stress response (ISR) is activated by a spectrum of cell damage-associated stimuli including protein misfolding in the endoplasmic reticulum (ER), protein aggregation in the cytosol, mitochondrial dysfunction, oxidative stress and amino acid deficiencies^1^. The two principal arms of the ISR include inhibition of general protein synthesis and induction of a transcriptional response that is mediated by several transcription factors including ATF4 and CHOP/DDIT3^1^. Both arms are activated by phosphorylation of the translation factor eIF2α that reduces general protein synthesis while increasing translation of select mRNAs such as *Atf4* or *Chop*^1^. ATF4 is the major mediator to the transcriptional arm of the ISR^1–4^. While ATF4 partners with CHOP to co-regulate many ISR-induced genes, it also directly stimulates *Chop* gene transcription^1–4^. The ATF4-driven gene expression program includes genes involved in restoration of cellular homeostasis such as components of the anti-oxidant defense systems, proteostasis, amino acid transport/synthesis and translation^2,5^. However, ATF4/CHOP-mediated upregulation of genes from the two latter categories may result in cytotoxicity^5,6^. Specifically, the resulting recovery from the ISR-mediated translational inhibition favors survival of cells with successful restoration of cellular homeostasis as increased translation under persistent stress becomes toxic^5,6^. In the case of persistent ER stress, nascent protein overload of the malfunctioning ER results in ER-based production of reactive oxygen species (ROS) and subsequent damage of the ER-associated mitochondria^5,6^. Such cytotoxicity may be further promoted by CHOP-regulated expression of pro-apoptotic genes^3^. Noteworthy, CHOP may also serve as a negative regulator of at least some ATF4-induced genes^2^. Therefore, CHOP plays a complex role in the transcriptional arm of the ISR by being an ATF4 partner in gene upregulation events, mediating a counterregulatory feedback to limit ATF4 activity, and also, reducing gene expression by inhibiting other transcription factors such as CEBPB^2^.

White matter damage after traumatic spinal cord injury (SCI) is associated with axonal degeneration, death of oligodendrocytes (OLs) and demyelination^7,8^. In contusive SCI at thoracic level, white matter damage is the main driver of SCI-associated loss of hindlimb function^9^. Thus, reducing OL loss is a major target for acute neuroprotection in these injuries. Various white matter pathologies such as SCI, experimental autoimmune encephalomyelitis (EAE) or some types of degenerative leukodystrophies are associated with activation of the transcriptional arm of the ISR in various neural cells including OLs, astrocytes, microglia, neurons, and microvasculature^10–16^. However, the outcome of such a response may vary dependent on the ISR mediator as well as the pathological and/or cellular context. In a mouse model of the degenerative leukodystrophy Pelizeus-Merzbacher disease (PMD), general *Chop* gene deletion (*Chop*^−/−^) exacerbated OL apoptosis and demyelination^15^. Moreover, while OL-specific overexpression of *Chop* did not cause any loss of those cells or abnormalities in myelin development, it had no modifying effects on white matter pathology in PMD mice^17^. In contrast, *Chop*^−/−^ mice showed reduced white matter damage and improved locomotor recovery after moderate contusive T9 SCI^11^.

A major limitation of experiments using general, all-cell null mutants of *Chop* or *Atf4* is the inability to directly identify the cell types in which those ISR mediators regulate white matter damage. After SCI, CHOP accumulates in OLs, but also in neurons, astrocytes and microvasculature^10,11,14^. Therefore, improved post-SCI survival of OLs in *Chop*^−/−^ mice may result from dampened ISR-associated transcription in OLs and/or other neural cells. Indeed, in vanishing white matter disease, in which ATF4 is activated in astrocytes and microglia, astrocytes drive white matter pathology by reducing OL maturation^16,18^. The current study used OL-*Atf4*^−/−^ and OL-*Chop*^−/−^ mice to determine whether OL activity of those principal mediators of the transcriptional arm of the ISR modulates white matter damage and functional recovery after contusive SCI.

## Results

### Transient versus persistent activation of the ATF4/CHOP-driven transcription in OLs after moderate contusive SCI

**Figure 1:**
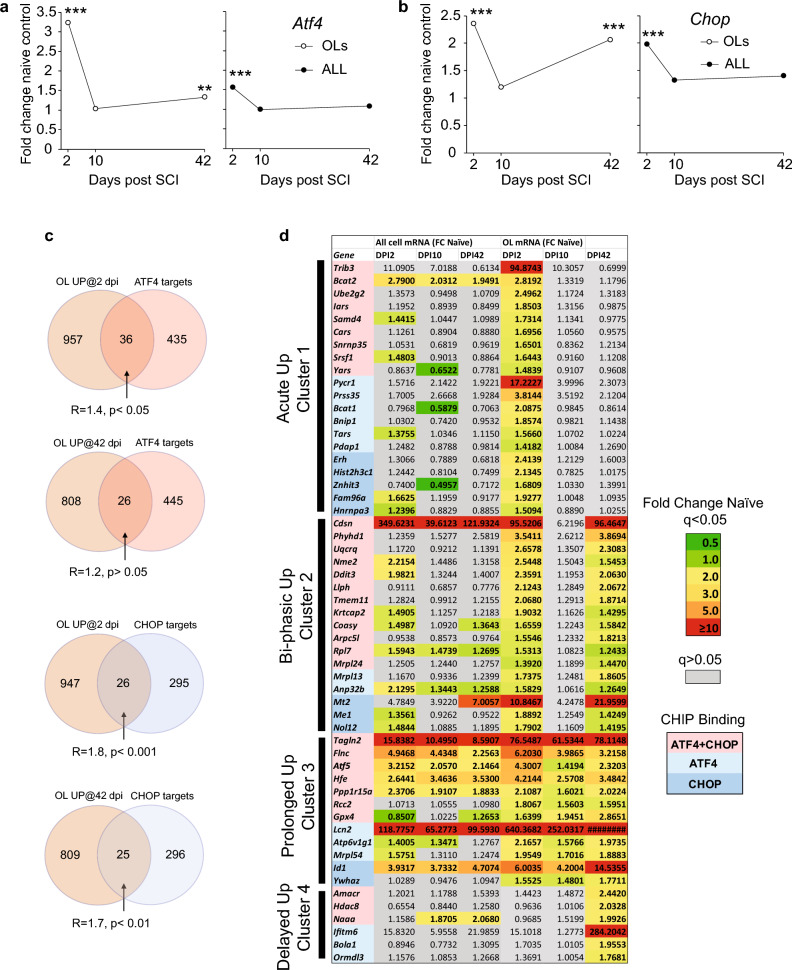
The transcriptional arm of the ISR is activated both acutely and chronically in OLs from the contused spinal cord tissue. Expression data were retrieved from all cell- or OL-enriched translatome datasets obtained from female OL-RiboTag mice by RNASeq. Mice whose ribosomes were tagged selectively in mature OLs received moderate contusive SCI (50 kdyn contusion, T9) and ribosome associated RNAs were isolated from spinal cord tissue including the injury site (all cell samples/input/). OL-enriched ribosomes and their associated mRNAs were then purified by RiboTag immunoprecipitation (OL samples/immunoprecipitated/). Data represent 3 independent samples, 2 animals/each. As compared to non-injured, naive OL-RiboTag mice, SCI animals showed increased expression of *Atf4* (**a**) and *Chop* (**b**) in OLs on dpi 2 and dpi 42, or, in all cells on dpi 2. (**c**) A list of direct ATF4/CHOP target genes that were identified by CHIPSeq in mouse cells with activated ISR^5^ showed moderate but significant overlaps with SCI upregulated OL genes from OL-RiboTag mice (genes with expression ≥ 1.41 fold uninjured controls, q < 0.05). The representation factor R is a ratio between the number of overlapping genes and the expected number of a random overlap between two samples of the identical size if drawn from 17,680 genes whose expression was detected in RiboTag samples, *p* values are for a hypergeometric test. (**d**) A heat map representing SCI effects on expression of OL-upregulated genes that are known as direct targets of ATF4 and/or CHOP. Over 90% of such genes clustered into one of the four kinetical patterns. Note that 30/45 ATF4 targets and 27/41 CHOP targets showed chronic OL upregulation at dpi 42. At that time, OL expression of at least two OL-upregulated ATF4/CHOP targets genes, ATF5 and GPX4 was confirmed by immunostaining (Supplementary Figs. S1 and S2).

Previous reports relied on CHOP immunostaining to show acute, SCI-associated activation of the transcriptional arm of the ISR in OLs^10,11^. However, neither duration nor transcriptomic scope of such a response was described. Therefore, we analyzed a SCI RNASeq dataset from OL-specific RiboTag mice to determine OL vs. total spinal cord tissue expression of *Atf4*, *Chop* as well as their downstream target gene transcripts. The dataset represented translatomes (*i.e.* polysome-associated mRNAs) from all cells (total RNA) or from mature OLs (OL RNA) that survived a moderate contusive T9 SCI (50 kdyn, IH). The tissue analyzed included a 5 mm spinal cord segment with the injury site in the center (see Methods for more details). Consistent with previous reports, total spinal cord expression of *Atf4* or *Chop* increased acutely 1.5- or twofold of uninjured controls at 2 days post-injury (dpi), respectively (Fig. 1, q < 0.001). Conversely, subacute (dpi 10) or chronic (dpi 42) expression of those ISR mediators was similar to controls (Fig. 1, q > 0.05). In OLs, both *Atf4* and *Chop* were upregulated on dpi 2 and dpi 42, but not dpi 10 (Fig. 1). Acutely, OL levels of *Atf4* or *Chop* reached 3.25- or 2.4-fold of controls, respectively (Fig. 1, q < 0.001). Chronically, OL *Chop* was up 2.1-fold of control (Fig. 1b, q < 0.001). The OL *Atf4* increase at that time was modest, but significant (1.3- fold of control, Fig. 1b, q < 0.001).

Next, the translatome datasets were analyzed to determine SCI effects on expression of genes directly regulated by ATF4 and/or CHOP in mouse fibroblasts with activated ISR^5^. Among OL upregulated genes (≥ 1.41-fold of control, q < 0.05), ATF4 targets were moderately overrepresented on dpi 2 but not dpi 10 or dpi 42 (Fig. 1c, 1.4-fold of the expected random overlap, p < 0.05). CHOP targets were overrepresented on dpi 2 and dpi 42, but not dpi 10 (Fig. dc, 1.7- or 1.8-fold overrepresentation on dpi 2 or 42, respectively, *p* < 0.01). Comparing the kinetics of SCI-associated expression changes, most of the OL-upregulated ATF4 targets (45 of 49 genes) clustered into one of 4 groups (Fig. 1d). While 15 genes were upregulated only on dpi 2 (cluster 1), 30 were also up on dpi 42 (clusters 2–4). Of them, 24 were upregulated at both timepoints, including 14 with a biphasic regulation (cluster 2, Fig. 1d) and 10 that were also up on dpi 10 (cluster 3, Fig. 1d). A similar clustering pattern was found for OL-upregulated CHOP targets (14/41 or 27/41 targets in cluster 1 or clusters 2–4, respectively, Fig. 1d). Co-immunostaining analysis using an antibody for the mature OL marker epitope CC1 confirmed OL expression of protein products of at least two ATF4/CHOP target genes that showed prolonged OL upregulation. Specifically, on dpi 42, signals for ATF5 or GPX4 were observed in CC1^+^ OLs from the spared white matter 1 mm rostral and caudal from the injury epicenter (Supplementary Figs. S1 and S2). While these data confirm acute upregulation of the transcriptional arm of the ISR in OLs, they also uncover unexpected persistence and/or re-activation of that pathway throughout the chronic phase of SCI recovery. As after contusive SCI in rodents, most OL loss occurs acutely with some OL apoptosis continuing subacutely^7,8^, chronic activation of OL ISR suggests its potential role in regulation of processes other than cell death. Those could include repair and/or re-organization of the white matter and/or maintenance of spared axon function.

### Limited effects of OL-specific knockouts of Atf4 or Chop on expression of OL marker mRNAs acutely after SCI

**Figure 2:**
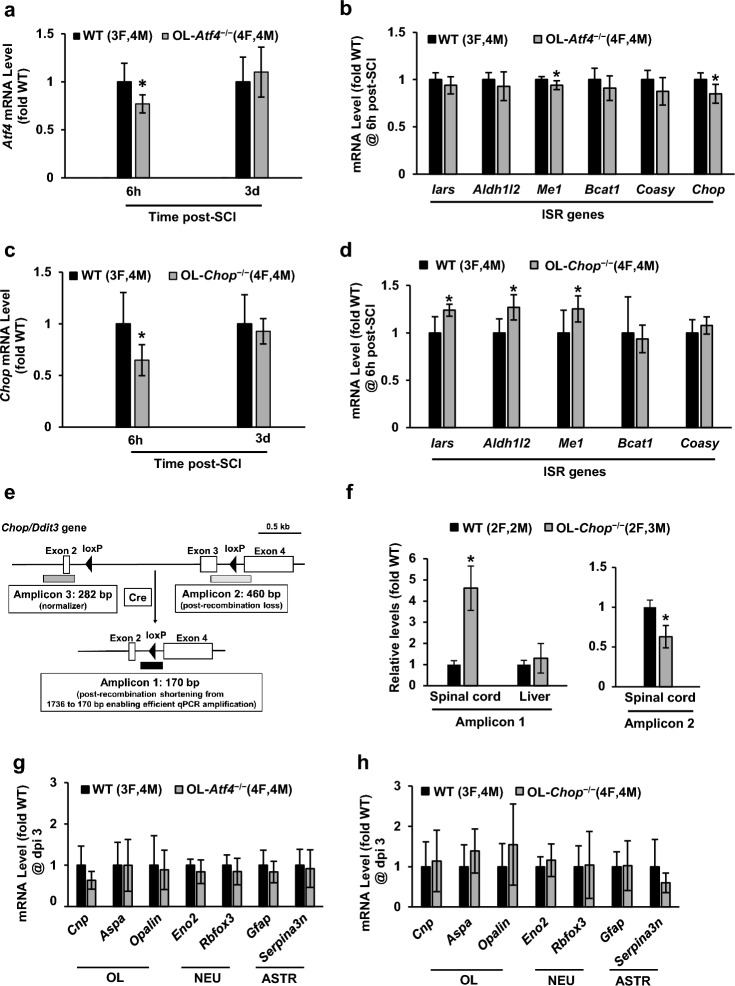
Unaffected levels of neural cell marker mRNAs in OL-*Atf4*^*−/−*^ or OL-*Chop*^*−/−*^ mice acutely after SCI (IH 50 kdyn, T9). (**a–d, g, h**), Total RNA was isolated from 5 mm spinal cord segments that were centered on the injury site and mRNA expression was determined by qPCR with *Gapdh* used as a normalizing transcript. At 6 h but not 3 days post-SCI, lower levels of *Atf4* (**a**) or *Chop* (**c**) were found in OL-*Atf4*^*−/−*^ or OL-*Chop*^*−/−*^ mice, respectively. These initial declines are consistent with efficient deletion of *Atf4* or *Chop* in OLs; wt-like expression on dpi 3 is likely due to altered cellular composition of the spinal cord tissue including loss of mature OLs and/or robust ISR activation in non-OL cells (see text for more details). (**b, d**), Expression of select ISR mRNAs whose genes are direct targets of ATF4 and/or CHOP at 6 h post SCI. Note significant effect of OL-*Atf4* or OL-*Chop* KOs on some of those transcripts. No significant changes in expression of any ISR genes were found on dpi 3 (Supplementary Fig. S3). (**e, f**), Genomic DNA was isolated from spinal cord or liver of naïve OL-*Chop*^*−/−*^ or wt mice at two weeks after completion of the tamoxifen knock out induction protocol and analyzed by qPCR. (**e**), Design of the qPCR assays to analyze Cre-mediated recombination of the *Chop* gene (see Supplementary Fig. S4 for additional amplicon validation data). (**f**), Efficient and specific recombination of the *Chop* gene in the spinal cord of OL-*Chop*^*−/−*^ mice. Note increased gene recombination and loss of the floxed exon 3 as detected by amplicons 1 and 2, respectively. (**g, h**), On dpi 3, unaltered expression of neural cell type marker mRNAs suggests unaffected loss of neurons and OLs as well as similar extent of reactive astrogliosis. Means ± SD are shown, animal numbers/sex are indicated (*, *p* < 0.05; ns, *p* > 0.05, Mann–Whitney *u*-test; M, males; F, females).

To determine an OL cell autonomous role of ATF4 and CHOP in SCI pathogenesis, mice with OL-selective deletion of *Atf4* (OL-*Atf4*^*−/−*^) or *Chop* (OL-*Chop*^*−/−*^) were used. Such mutations were generated by tamoxifen treatment (1 mg *i.p.* daily for 8 days) of double transgenic mice that combined the *Plp1* promoter-driven *Cre*^*ERT2*^ recombinase with floxed alleles of *Atf4* or *Chop*^19–21^. Littermates of OL-*Atf4*^*−/−*^ or OL-*Chop*^*−/−*^ mice that received vehicle instead of tamoxifen were used as wild type (wt) controls. Two weeks after the last tamoxifen injection, SCI was performed (IH 50 kdyn at T9) and mRNA expression of *Atf4*, *Chop* as well as their select target genes was evaluated at 6 or 72 h (dpi 3). This gene deletion verification study was limited to injured mice as in the intact CNS, basal expression of ISR genes including *Atf4* or *Chop* is expected to be low. At 6 h after SCI, *Atf4* or *Chop* mRNAs decreased by about 20% in OL-*Atf4*^*−/−*^ or OL-*Chop*^*−/−*^ mice, respectively (Fig. 2a, c, p < 0.05). That early reduction is consistent with the estimated 20–25% contribution of mature OLs to the total spinal cord cellularity^22,23^ and the SCI-associated ISR activation in all major types of neural cell^10,11^. In addition, efficient, OL-specific KO of *Atf4* and *Chop* is supported by similar declines in total spinal cord expression of other broadly expressed genes deleted in OLs using the *Plp1-Cre*^*ERT2*^ line^24^. However, on dpi 3, total spinal cord expression of *Atf4* or *Chop* was unaffected by their respective OL-KOs (Fig. 2a,c, p > 0.05). Such findings are likely due to SCI-induced changes of spinal cord cellular composition and/or high levels of ISR activation in non-OL cells at later times post-injury. Thus, on dpi 3, a reduced content of mature OLs and increases in oligodendrocyte precursor cells (OPCs), vascular cells and innate immune cells coincides with ISR activation in those expanding cell populations^7,8,25–27^. Therefore, on dpi 3, altered ISR activity in surviving OLs from OL*-Atf4* or OL*-Chop* null mice may be hard to detect in total spinal cord RNA samples. Consistent with such interpretation, select ATF4 and/or CHOP target gene transcripts were also affected by OL-*Atf4* or OL-*Chop* deletion at 6 h, but not 3 days post SCI (Fig. 2b,d and Supplementary Fig. S3). Thus, *Chop* and *Me1* mRNAs were significantly decreased in OL-*Atf4*^*−/−*^ mice (Fig. 2b). Conversely, *Iars*, *Aldhl12* and *Me1* were upregulated in OL-*Chop*^*−/−*^ mice (Fig. 2d). Therefore, in OLs, CHOP may be a negative regulator of at least some ISR genes.

Importantly, at least for OL*-Chop*^*−/−*^ mice, efficient and specific deletion of the floxed exon 3 of the *Chop* gene was also confirmed by qPCR analysis of genomic DNA (Fig. 2e,f, Supplementary Fig. S4). For those assays, spinal cord or liver DNA was isolated from naïve mice 2 weeks after completion of the tamoxifen induction protocol. As compared to vehicle-treated littermate controls (wt), OL-*Chop*^*−/−*^ spinal cord DNA showed a 4.6-fold increase in Cre-dependent recombination inside the *Chop* gene that was accompanied by a 38% loss of the floxed exon 3 (Fig. 2f, p < 0.05). Conversely, no change in *Chop* gene recombination was observed in OL*-Chop*^*−/−*^ versus wt liver where OLs are absent. Of note, in the spinal cord of wt mice, there was low, but noticeable, baseline recombination of the *Chop* gene that is likely related to tamoxifen-independent activity of Cre-ERT2 (Supplementary Fig. S4). Such activity has been reported before for various CreERT2 mouse lines^28,29^. Given similar effects of OL-*Chop* or OL-*Atf4* knock out on mRNA expression of the respective floxed gene (*i.e. Chop* or *Atf4*, respectively, Fig. 2a,c), one can expect that Cre-mediated deletion of *Atf4* is as efficient and specific as that observed for *Chop*.

On dpi 2–3, declining expression of OL marker transcripts in the contused spinal cord tissue is a correlate of acute OL loss and SCI-associated white matter damage^30^. However, on dpi 3, the OL mRNAs *Aspa*, *Opalin*, or *Cnp* showed similar expression in wt vs. OL-*Atf4*^*−/−*^ or OL-*Chop*^*−/−*^ mice (Fig. 2g,h). Therefore, SCI-induced acute loss of OLs seems to be unaffected by OL-selective deletion of *Atf4* or *Chop*. Likewise, neither neuronal loss nor reactive astrogliosis appeared to be altered as expression of neuronal or reactive astrocyte marker transcripts was similar between OL-KOs and their respective wt controls at least on dpi 3 (Fig. 2g,h). Therefore, OL-selective loss of *Atf4* or *Chop* does not appear to modulate acute loss of OLs or neurons.

### Delayed modulation of functional recovery after SCI in OL-Atf4^−/−^ and OL-Chop^−/−^ mice

**Figure 3:**
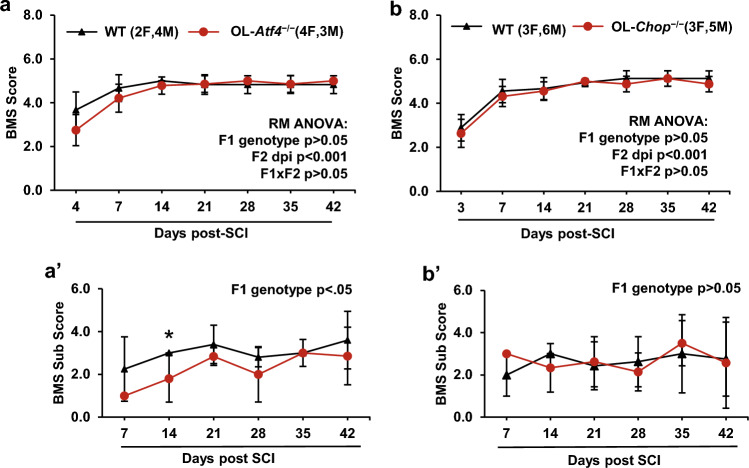
Unaffected BMS hindlimb function recovery in OL-*Atf4*^*−/−*^ or OL-*Chop*^*−/−*^ mice following moderate contusive SCI. BMS was analyzed after SCI (IH 50 kdyn, T9) as indicated. (**a, b**) Both wt and OL-KOs showed significant BMS recovery over the 6 week post-SCI period but no significant effect of genotype was detected (RM-ANOVA, factor 2 /F2: dpi/ vs. factor 1 /F1: genotype/). (**a′**) On dpi 14, OL-*Atf4*^*-/-*^ mice had significantly lower BMS subscore than wt controls. (**b′**) BMS subscores in OL-*Chop*^*-/-*^ mice did not differ from wt controls. Means ± SD are presented (*, *p* < 0.05 RM-ANOVA and Bonferroni post-hoc test); detailed results of RM ANOVA analysis are in supplementary Table S3.

**Figure 4:**
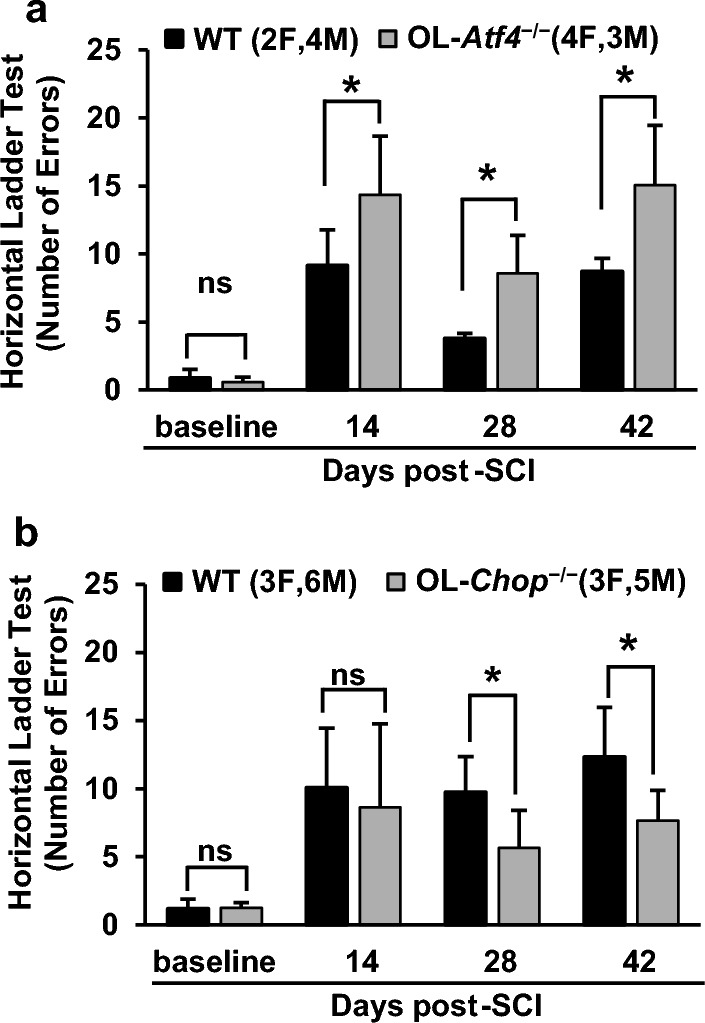
Opposing effects of OL-*Atf4* and OL-*Chop* knockouts on ladder test performance. Number of errors during horizontal ladder walking was analyzed before (baseline) and after SCI (IH 50 kdyn, T9) as indicated. (**a**) OL-*Atf4*^*−/−*^ mice made significantly more errors than wt controls on dpi 14, 28 and 42. (**b**) Number of errors was significantly reduced in OL-*Chop*^*−/−*^ mice vs. wt controls on dpi 28 and 42. For each testing session/genotype, a mean number of errors ± SD is shown (* or ns, *p* < 0.05 or > 0.05 RM ANOVA and Bonferroni post-hoc test, respectively; detailed results of RM ANOVA analysis are in supplementary Table S3).

**Figure 5:**
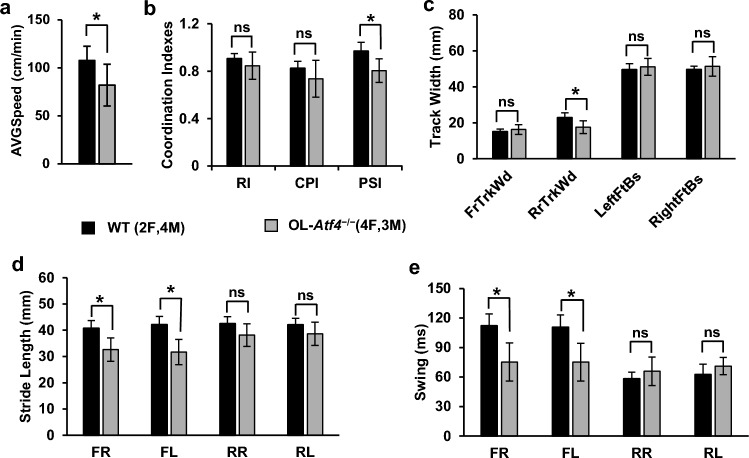
At 6 weeks post-SCI, gait analysis reveals worsened locomotor recovery in OL-*Atf4*^*−/−*^ mice. Analysis of gait was performed using the Treadscan system. For each mouse, an optimal walking speed was identified (maximal walking speed with consistent plantar stepping) at which gait parameters were measured. (**a**) OL-*Atf4*^*−/−*^ mice showed lower mean optimal walking speed. (**b**) Significant reductions of the plantar stepping index (PSI, plantar step number of hindlimbs to forelimbs) suggest impaired forelimb/hindlimb coordination. Four-limb stepping pattern indices are unaffected (the regularity index, RI, or the coordinated pattern index, CPI that show a fraction of correct stepping patterns of all 4 limbs including or excluding dorsal steps, respectively). (**c**) Rear track width (RrTrkWd) is significantly shorter. Other track parameters are unaffected (front track width, FrTrkWd; left/right foot base L/R FtBs). (**d, e**) Significantly shorter stride length and swing time suggest increased compensatory use of forelimbs in OL-*Atf4*^*−/−*^ mice (FR/FL, front right/left; RR/RL, rear right/left). For each gait parameter, a mean ± SD is shown; * or ns, *p* < 0.05 or > 0.05, *t*-test in (**a–c**) or ANOVA/Bonferroni post-hoc tests in (**d, e**) (detailed results of the statistical analysis are in supplementary Table S4). No significant effects on gait were found in OL-*Chop*^*−/−*^ mice after SCI (Supplementary Fig. S5).

After SCI, locomotor recovery was evaluated over a 6-week period using the BMS on dpi 3, 7 and then weekly afterwards), horizontal ladder walking (dpi 14, 28, and, 42), and Treadscan^®^ gait analysis (dpi 42). For BMS and ladder walking, baseline values were recorded before SCI but showed no significant differences between wt and OL-KOs. After SCI, BMS revealed strong improvement of hindlimb function over time, but no significant effects of either OL-*Atf4*^*−/−*^ or OL-*Chop*^*−/−*^ genotype (Fig. 3, see Supplementary Table S3 for detailed results of statistical analyses). Moreover, BMS subscores of these OL-ISR mutants did not differ substantially from wt controls except a significant decline in OL-*Atf4*^*−/−*^ mice on dpi 14 (Fig. 3). Significant differences emerged in performance of the horizontal ladder test. On dpi 14, 28, or 42 OL-*Atf4*^*−/−*^ mice did 57, 126, or 73% more ladder crossing errors than wt controls, respectively (Fig. 4a, p < 0.05, see Supplementary Table S3 for detailed results of statistical analyses). Conversely, 42 or 38% less errors were observed in OL-*Chop*^*−/−*^ mice on dpi 28 and 42 but not on 14, respectively (Fig. 4b, p < 0.05). Finally, on dpi 42, gait analysis using the Treadscan^®^ system uncovered significant changes in locomotor recovery of OL-*Atf4*^*−/−*^, but not OL-*Chop*^*−/−*^ mice (Fig. 5, Supplementary Fig. S5). Those included lower average speed of walking with plantar stepping, reduced ratio of hindlimb plantar stepping cycles to forelimb plantar stepping cycles (plantar stepping index, PSI), shortening of the rear track width as well as shortening of forelimb stride length and swing time (Fig. 5, *p* < 0.05, see Supplementary Table S4 for detailed results of statistical analyses). Those latter effects suggest increased compensatory usage to cover for greater impairment of hindlimbs. Given the indirect evidence of greater hindlimb dysfunction as suggested by lower walking speed and increased use of forelimbs, significant narrowing of the rear track suggests reduced balance control in OL-*Atf4*^*−/−*^ animals (Fig. 5c, p < 0.05). Such a deficit, together with decreased forelimb-hindlimb coordination (lower PSI), could have contributed to worsened ladder test performance of those mice after SCI. In addition, proficient ladder walking requires trunk stability, sensory feedback and control of the digits^31^. Hence, the opposing ladder walking phenotypes of OL-*Atf4*^*−/−*^ or OL-*Chop*^*−/−*^ mice may reflect distinct modulatory effects on such modalities of fine locomotor control.

As all those functional analyses included mice of both genders, additional statistical analyses were performed that included gender as a factor in ANOVA design. However, none of the recovery parameters analyzed showed a significant effect of gender (*e.g.* for OL-*Atf4*^*−/−*^ study, ladder test errors: F = 0.10, df = 1,25, *p* = 0.749, and BMS: F = 0.01, df = 1,37, *p* = 0.932; for OL-*Chop*^*−/−*^ study, ladder test errors: F = 0.02, df = 1,37, *p* = 0.898 and BMS: F = 2.9, df = 1,30, *p* = 0.10). However, as low numbers of animals were used, the gender effect analysis is underpowered. Therefore, potential gender-specific modulation of post-SCI recovery by the transcriptional arm of the OL-ISR should be addressed by future studies.

### SCI-induced white matter injury is unaffected in OL-Atf4^−/−^ and OL-Chop^−/−^ mice

**Figure 6:**
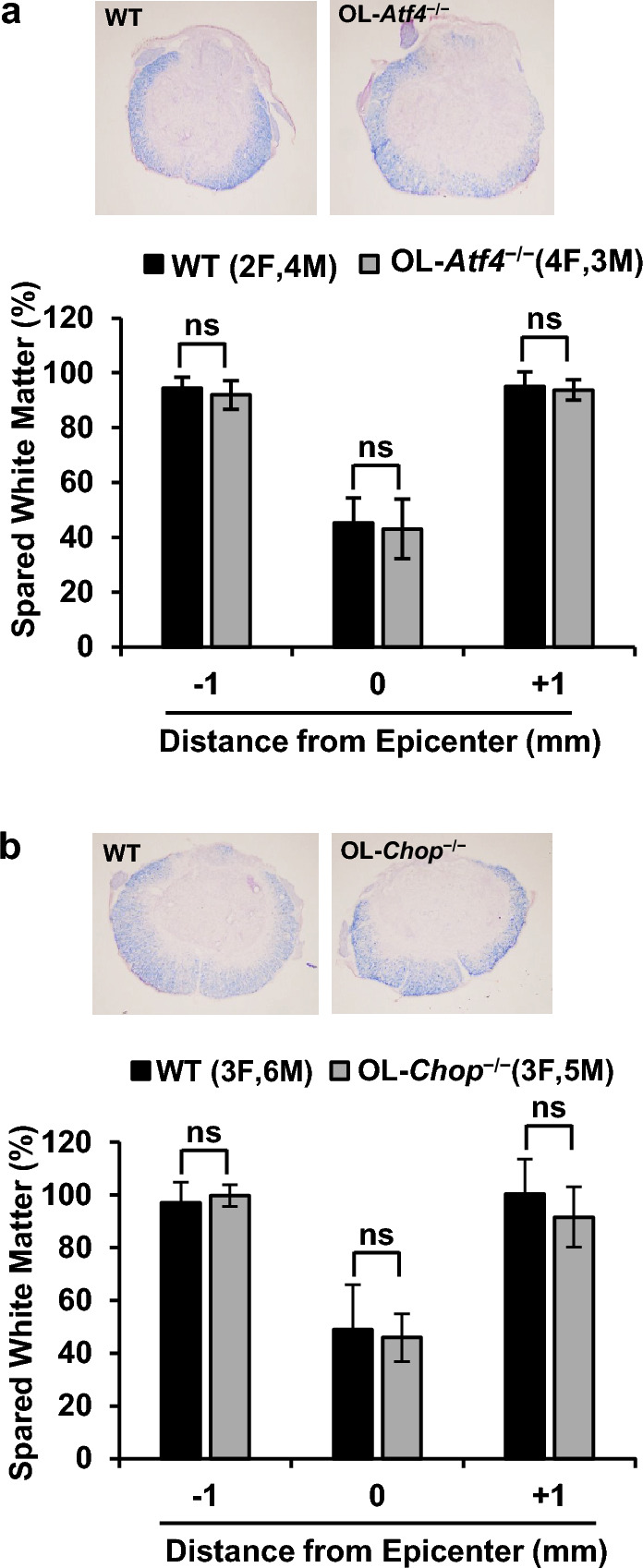
Unaffected white matter sparing at the injury epicenter of OL-*Atf4*^*−/−*^ or OL-*Chop*^*−/−*^ mice. Six weeks after contusive SCI (IH 50 kdyn, T9) mice were killed and white matter sparing (WMS) was evaluated on transverse sections using EC staining for myelin. Representative images of EC stained sections through the injury epicenter are shown. For each animal, WMS was calculated as EC^+^ (myelinated) white matter area/total section area and normalized to EC^+^ white matter area/total section area at 2 mm rostrally from the injury epicenter (white matter content in that region was set as 100%). At the injury epicenter, spared white matter content declined for each genotype as compared to sections at 1 mm rostrally or caudally to that region (*p* < 0.001, RM-ANOVA and Bonferroni post-hoc test). However, no significant effects of OL-*Atf4* (**a**) or OL-*Chop* (**b**) KO were observed present in any of those areas (ns, *p* > 0.05, see Supplementary Table S5 for detailed results of the statistical analysis).

On dpi 42, white matter sparring was analyzed using EC staining of myelin. At the injury epicenter, 70–75% of the myelinated white matter was lost in wt mice (Fig. 6, *p* < 0.05, see Supplementary Table S5  for detailed results of statistical analyses). Similar damage was observed in OL-KOs (Fig. 6, *p* > 0.05). In addition, OL-KOs showed unaffected white matter content 1 mm rostrally or 1 mm caudally from the epicenter (Fig. 6, *p* > 0.05). As the epicenter loss of myelinated white matter is a key correlate of hindlimb function that is determined by BMS^9^, these histological results are consistent with unaffected BMS scores in OL-*Atf4*^*−/−*^ or OL-*Chop*^*−/−*^ mice.

**Figure 7:**
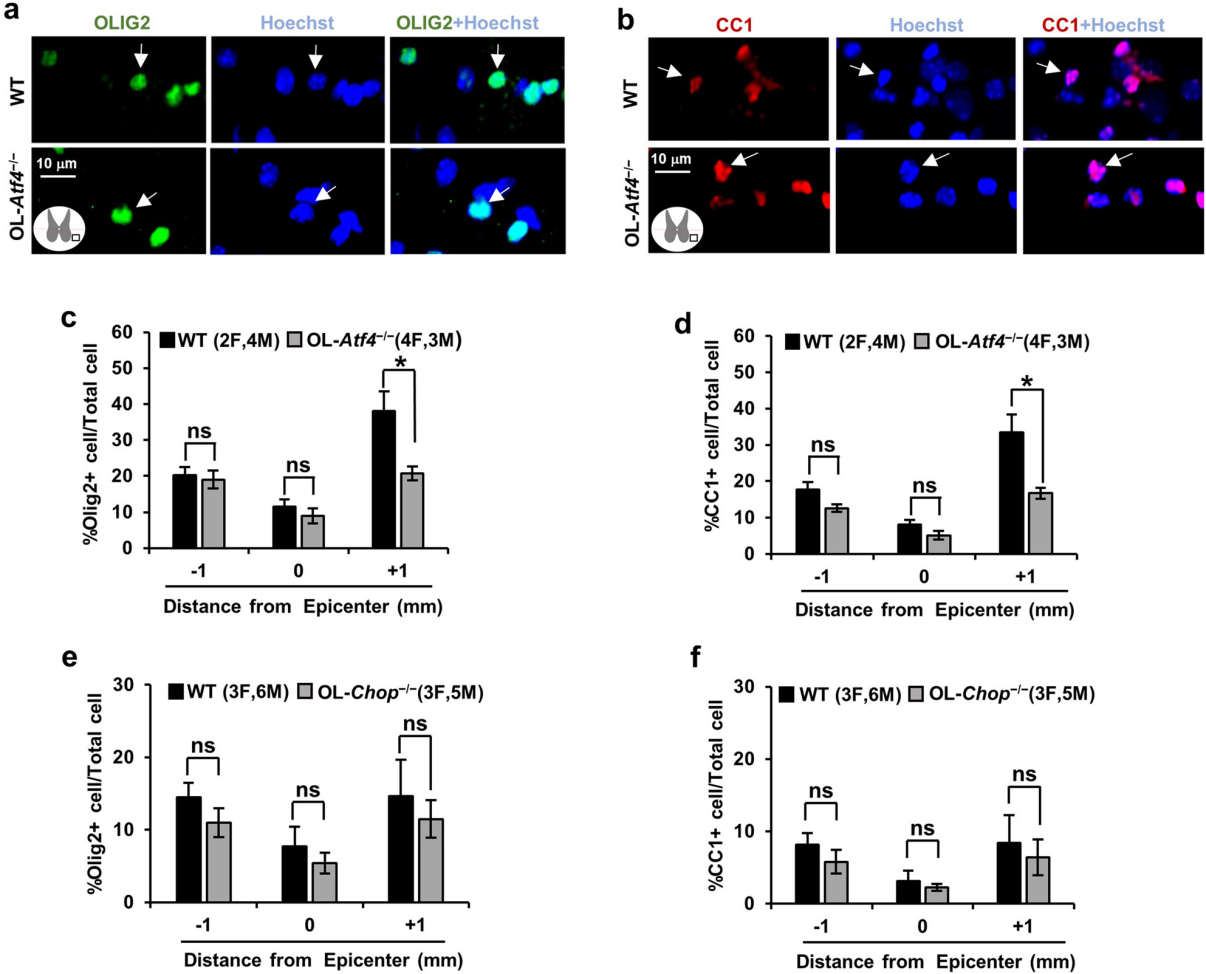
Unaffected OL content in the spared ventral matter at the injury epicenter of OL-*Atf4*^*−/−*^ or OL-*Chop*^*−/−*^ mice. Immunostainings for the OL lineage cell marker OLIG2 or the mature OL marker (CC1) were performed using spinal cord tissue collected on dpi 42 as described for Fig. 6. Representative images of OLIG2 (**a**) and CC1 (**b**) immunostainings in the spared ventral white matter of wt- or OL-*Atf4*^*−/−*^ mice at the injury epicenter; cell nuclei are counterstained with Hoechst-33258. Cells with positive immunostaining signals are indicated by arrows. The graphs present relative content of OL lineage cells (OLs and OPCs, OLIG2^+^/all cells) (**c, e**) or mature OLs (CC1^+^/all cells) (**d, f**) in the spared ventral white matter. For each genotype, OL/OPC content declined significantly at the injury epicenter as compared to Sections 1 mm rostrally or caudally from that region (RM ANOVA and Bonferroni posthoc, *p* < 0.001). Significant effects of a genotype were only found in OL-*Atf4*^*−/−*^ mice 1 mm caudally from the injury epicenter. Means ± SD are shown (*, *p* < 0.05; ns, *p* > 0.05 RM ANOVA/ Bonferroni post-hoc test, see Supplementary Table S5 for detailed results of the statistical analysis).

Finally, the relative content of all OL-lineage cells (OPCs and OLs) or mature OLs was analyzed in the spared, ventral white matter using OLIG2 or CC1 immunostaining, respectively (Fig. 7a,b). In all genotypes, OL/OPC content was lower at the injury epicenter than 1 mm rostrally or caudally from that region (Fig. 7c–f, p < 0.05; see Supplementary Table S5 for detailed results of statistical analyses). These findings are consistent with a lasting OL loss in the spared white matter at the epicenter level following contusive SCI^7,8^. However, the epicenter OL/OPC content was unaffected by the OL-*Atf4*^*−/−*^ or OL-*Chop*^*−/−*^ genotype (Fig. 7c–f, p > 0.05). Interestingly, as compared to wt, OL-*Atf4*^*−/−*^ mice showed reduced OL/OPC content in the ventral white matter 1 mm caudally, but not rostrally, to the epicenter (Fig. 7c,d, p < 0.05). No such effects were present in OL-*Chop*^*−/−*^ mice (Fig. 7e,f). Overall, these histological analyses support a notion that at the injury epicenter, the lasting loss of spared white matter OLs is not affected by OL-selective deletion of *Atf4* or *Chop*. Outside of that region, lost OLs are effectively replaced by new OLs that are produced by OPCs with OL content recovering to or above pre-injury levels by 4–5 weeks after contusive SCI^7,8^. Therefore, the lower caudal OL content in OL-*Atf4*^*−/−*^ mice may reflect reduced generation and/or survival of new OLs. Alternatively, pre-existing OLs in the caudal white matter may be selectively dependent on ATF4 for survival in the injured spinal cord environment. Regardless of a mechanism, the functional significance of altered OL content in the caudal region of the spared ventral white matter is unclear.

## Discussion

Current results reveal OL-cell autonomous roles of ATF4 and CHOP in functional recovery post-SCI. Unexpectedly, those roles are opposite and likely unrelated to a lasting loss of OLs or white matter sparing at the injury epicenter, as there were no apparent differences in latter processes between the two strains. Interestingly, the worsening or improvement of fine locomotor control in OL-*Chop*^*−/−*^ or OL-*Atf4*^*−/−*^ mice, respectively, persists through the chronic phase of recovery correlating with a delayed OL upregulation of ATF4/CHOP target genes. Therefore, chronic activation of the OL-ISR may modulate function of neuronal circuitries that mediate post-SCI recovery.

ATF4, and particularly CHOP, are often implicated as cell autonomous-mediators of stress-induced death and/or survival of neurons and OLs^3,11,15,32–36^. Therefore, we anticipated that OL-selective deletions of *Atf4* and/or *Chop* will modulate SCI-associated acute death of OLs as well as their lasting loss and white matter damage at the injury epicenter. Instead, our data suggest that those pathological processes do not require OL-cell autonomous activities of ATF4 or CHOP.

While general *Chop* deletion has strong effects on OL loss, white matter sparing and functional recovery post-SCI^11^, OL-*Chop*^*−/−*^ mice show functional recovery improvements that could be detected only on more complex sensorimotor tasks. Moreover, no significant effects on acute OL loss or chronic white matter sparring were found in OL- *Chop*^*−/−*^ mice. Such differences suggest that CHOP activity in cells other than OLs is a positive regulator of acute white matter damage that, in turn, determines loss and recovery of hindlimb function. It is possible that those cells include microglia and/or monocyte-derived macrophages, which are key drivers of the cytotoxic neuroinflammation post-SCI^26,37^. Reduced macrophage-mediated inflammation has been reported in non-CNS injury models in *Chop*^*−/−*^ mice or in *Chop*^*−/−*^ macrophages in culture^38–41^. Therefore, targeting CHOP after SCI may exert pleiotropic benefits across several cell types including attenuation of microglia/macrophage-mediated white matter damage as well as enhancement of OL support for neuronal circuitries controlling fine locomotor control.

Although no contusive SCI data from *Atf4*^*−/−*^ mice have been reported, such animals showed worsened locomotor BMS recovery following T12 hemisection SCI^42^. Attenuated increases in OL lineage cell numbers were also found^42^. Therefore, as compared to the current findings from OL- *Atf4*^*−/−*^ mice, the SCI phenotype of the general *Atf4* KO shows at least similar direction with respect to functional recovery and OL linage cell content.

Besides cell death and survival, the ISR regulates other aspects of cellular physiology including metabolism, stress adaptation, and cell–cell communication^1–4^. In neurons, the ISR may modulate the function of neuronal circuitries, including neuroplasticity and cognition^43,44^. Thus, persistent neuronal activation of the ISR kinase PERK/EIF2AK3 has been implicated as a driver of impaired cognition in a mouse model of Alzheimer’s disease (AD)^45^. Moreover, chronic activation of the ISR was found in mouse traumatic brain injury (TBI) and its pharmacological attenuation improved cognitive recovery^46^. Intriguingly, the current data suggest that in surviving OLs, the transcriptional arm of the ISR is activated chronically after SCI. In addition, SCI phenotypes of OL-*Atf4*^*−/−*^ and OL-*Chop*^*−/−*^ mice support a possibility that such a response regulates fine motor control during locomotor recovery in an ISR mediator-specific manner. Those findings raise an interesting possibility that the functional outcomes of prolonged ISR activation following CNS injury are determined by a stressed cell type and/or an ISR mediator.

What could be the potential mechanisms behind functional modulation of the fine locomotor control downstream of OL-ISR? One possibility is that the ISR in surviving OLs modulates remyelination. Post-SCI remyelination is mediated by new OLs that are generated from OPCs in response to injury^7,8^. Post-SCI oligodendrogenesis may lead to higher OL number especially in areas distal to the lesion site^47^. Hence, lower content of OLs/OL lineage cells in OL-*Atf4*^*−/−*^ mice selectively at + 1 mm from the epicenter could suggest a location-specific and potentially non-cell autonomous requirement of OL ATF4 in OPC-mediated oligodendrogenesis including proliferation, differentiation and/or survival of newly generated OLs. In addition, pre-existing OLs may be capable of remyelination^48,49^. Hence, the transcriptional arm of the OL-ISR could also modulate this reparative process in a cell-autonomous manner. However, significance of post-SCI remyelination is unclear and studies using a mouse mutant that was not capable of remyelination suggests no major role of that process in functional recovery^50^. Current data are consistent with a limited role of OL-mediated remyelination in post-SCI recovery of hindlimb function, at least in a mouse model of contusive SCI with BMS as major functional readout.

In addition, OL ISR may modulate recovery of fine motor control by altering functional properties of spared myelin sheaths that wrap intact axons^51,52^. Both ATF4 and CHOP are well positioned to exert a broad control over cellular metabolism and potentially regulate OL function and/or OL-mediated support of axons. ATF4 is a positive regulator of a large group of genes involved in protein- and amino-acid synthesis^2,5,6^. Lipid synthesis is another critical component of myelin biogenesis and maintenance that may be positively regulated by ATF4^53–55^. Moreover, ATF4 is a positive regulator of the lactate generating anerobic glycolysis, which is highly active in OLs to support axonal oxidative metabolism^56,57^. Therefore, ATF4 may not only support macromolecule synthesis that is required for repair and/or maintenance of myelin but may also drive OL production of axon supporting metabolites such as lactate.

As many target genes of ATF4 are co-regulated by CHOP, it may seem surprising that the functional recovery phenotypes of OL-*Atf4*^*−/−*^ and OL-*Chop*^*−/−*^ mice are mostly opposite. However, the ATF4/CHOP co-regulation data come from cultured cells whose ISR was activated by ER stress-inducing agents such as tunicamycin or thapsigargin^5^. In other contexts, including whole animal tissues and/or other ISR-inducing stimuli, CHOP may oppose transcription that is mediated by ATF4, including upregulation of pro-anabolic genes that support amino acid supply and protein synthesis^58,59^. Importantly, increased expression of select ISR-associated transcripts in OL-*Chop*^*−/−*^ mice supports a CHOP-mediated feedback that restricts ATF4 activation in SCI-challenged OLs. Such feedback could explain opposite SCI recovery phenotypes in OL-*Chop*^*−/−*^ and OL-*Atf4*^*−/−*^ mice.

Together, current data suggest that in OLs, the transcriptional arm of the ISR that is mediated by ATF4/CHOP is not a major regulator of acute OL loss after SCI. Instead, it regulates fine motor control during later stages of the recovery correlating with its delayed activation in OLs. While OL-ATF4 promotes, OL-CHOP reduces that recovery. Therefore, enhancing activity of ATF4 and/or inhibiting CHOP offers potential strategies to improve functional outcome after SCI. The therapeutic window for such strategies may extend into the subacute and/or chronic phases of the recovery increasing their translational potential.

## Materials and methods

### Animals

The following mouse strains were obtained from the Jackson laboratories: C57Bl6, B6J.129(Cg)-*Rpl22*^*tm1.1Psam*^/SjJ (RiboTag^+/wt^, Strain # 029,977) B6.Cg-*Ddit3*^*tm1.1Irt*^/J (*Chop *^*fl/fl*^*,* Strain #030,816)*,* B6.Cg-Tg(Plp1-cre/ERT)3Pop/J (*Plp-cre*^*ERT2*^*,* Strain # 005975). The *Atf4 *^*fl/fl*^ mice with *loxP* sites flanking *Atf4* exons 2–3 were generously provided by Dr. Christopher Adams (University of Iowa)^20^. The following mice were generated by crossing those parental strains: RiboTag:*Plp-cre*^*ERT2*^ (both transgenes at heterozygosity), *Atf4*^*fl/fl*^:*Plp-cre*^*ERT2*^ and *Chop *^*fl/fl*^*:Plp-cre*^*ERT2*^ mice (the *Plp-cre*^*ERT2*^ transgene at homozygosity). To induce OL-selective ribosome labeling (removal of a stop codon to produce HA-tagged Rpl22 in RiboTag mice) or deletions of *Atf4*^*fl/fl*^ or *Chop*^*fl/fl*^ loci, 6–8 week old mice of the respective genotypes received 8 daily i.p. injections of tamoxifen (1 mg/day)^24,60^. Littermates that received vehicle injection (sunflower oil) were used as wild type (wt) controls. Experiments using OL-RiboTag, OL-*Atf4*^*−/−*^, or OL-*Chop*^*−/−*^ mice started 2 weeks (OL-ISR KOs) or 30 days (OL-RiboTag) after the last tamoxifen injection. Non-transgenic, female C57Bl6 mice were used for immunostaining analyses of ATF5 or GPX4 and as additional controls for validation of the *Chop* gene recombination assays. All animal experiments were approved by the University of Louisville Institutional Animal Care and Use Committee and Institutional Biosafety Committee and strictly adhered to NIH guidelines on use of experimental animals. In addition, ARRIVE guidelines were followed including random order of SCI or sham surgeries in animals from different experimental groups. However, fully randomized design of experimental groups was not possible due to limited availability of animals with required genotypes. Therefore, non-random group assignments were needed to obtain sufficient animal number for adequate statistical power and ensure balanced inclusion of both sexes.

### Materials

Antibodies were obtained from commercial suppliers as indicated. All other reagents and materials were purchased from Sigma Aldrich (St. Louis, MO), VWR International (Radnor, PA), Thermo Fisher Scientific (Waltham, MA), or Integrated DNA Technologies (IDT, Coralville, IA).

### Spinal cord injury

 SCI was performed as described previously^11^. Briefly, 8–10 week old mice were anaesthetized by an i.p. injection of 400 mg/kg 2,2,2-tribromoethanol. Lacri-Lube ophthalmic ointment (Allergen, Irvine, CA) was applied to prevent drying of eyes. After a laminectomy at the T9 vertebral level, moderate contusion injuries (50 kdyn force/400–800 μm displacement) were performed at using the IH impactor (Infinite Horizons, Lexington, KY). Postoperative care included s.c. 0.1 ml saline (immediately after surgery), 0.1 mg/kg s.c. buprenorphine every 12 h for 2 days, and manual expression of bladders twice a day for seven to ten days or until spontaneous voiding returned. All surgical and post-operation procedures were completed according to NIH and IACUC guidelines.

### Hindlimb locomotor function

 Hindlimb function was evaluated in an open field using the Basso Mouse Scale (BMS)^61^. Evaluations were performed at baseline before the injury and weekly thereafter. All raters were trained by Dr. Basso and colleagues at the Ohio State University and were blinded to genotype or surgical identity. The horizontal ladder test was performed as described previously using Columbus Instruments Sensor and RS-232 Mini Counter (Columbus Instruments; Columbus, OH, USA, 2.5 mm rungs spaced 3.5 cm apart)^62^. Briefly, each animal underwent five stepping trials per session and the total number of footfalls was quantified for the left and right limbs, respectively. A baseline session before SCI was followed by bi-weekly assessments starting at 2 weeks post-injury. Gait was analyzed using the Treadscan^®^ gait analysis system (Cleversys, Reston, VA) after completion of all locomotor assessments at week 6 after SCI as described previously^63^. Briefly, mice were placed on a variable speed treadmill with a clear belt, and a high-speed digital video (ventral view) of the stepping animals was recorded. Each animal was placed on the treadmill, the speed of the belt increased until the animal could no longer maintain position, and then decreased slowly until stable walking was achieved. Ten + consecutive step cycles were recorded at such individually optimized walking speed (if an animal was unable to execute 10 cycles, at least two 5 +  step cycles were recorded). A minimum of 8 step cycles/animal were then analyzed using the Treadscan software.

### Tissue collection and processing

Mice were deeply anaesthetized using 2,2,2-tribromoethanol and perfused transcardially with ice cold phosphate buffered saline (PBS, 30 ml/mouse). For RNA analysis, spinal cord samples (5 mm centered on the injury epicenter) were collected and frozen in liquid nitrogen. Samples for genomic DNA isolation were collected and stored in a similar manner. For histology, mice were perfused with 4% paraformaldehyde (PFA, pH 7.4 in PBS). Dissected spinal cords were post-fixed (4% PFA for 1 h at 4 °C), cryoprotected overnight in 30% sucrose (pH 7.4, 72 h at 4 °C) and mounted in TFM tissue freezing medium (GeneralData, Cincinnati, OH). Cryostat Sections (20 μm) were cut, thaw mounted on slides and stored at − 20 °C until further use.

### Translatome analysis using RiboTag mice

After SCI, female OL-RiboTag mice were anesthetized and transcardially perfused with ice cold PBS. Dissected spinal cords (5 mm spanning the injury epicenter) were flash frozen in liquid nitrogen and stored at − 80 °C until further use. Two frozen SCI epicenter samples from SCI or naïve animals were pooled to produce one biological replicate (n = 3/group, 6 animals/group). SCI and naïve samples were processed for input polysome-associated mRNAs (IN) or immune-precipitated (IP) OL polysome mRNAs as previously described^64^. RNA isolation, library preparation and RNA sequencing on the Illumina NextSeq 500 platform were performed following standard procedures. *Following* Fast QC quality control, the reads were directly aligned to the *Mus musculus* reference genome assembly (mm10.fa) using STAR (version 2.6) followed by raw read count (HTSeq version 0.10.0). The number of raw reads/sample was 36,812,296 to 39,546,186 (average 37,495,614). At least 96.56% reads were aligned to the mouse reference genome (average read alignment 98.06%). The raw counts were normalized using the Relative Log Expression (RLE) method and then filtered to exclude genes with fewer than 10 counts across samples. Differential expression was analyzed using DESeq2. Pairwise differential expression for IN or IP comparing SCI samples to naïve samples was performed to identify changes in gene expression after SCI. Differential expression between IP and IN was also analyzed for each set of samples to determine OL-enriched expression in naïve or SCI mice. False discovery rate-corrected p-value (q) was calculated for each transcript with q < 0.05 to identify significant gene expression changes. In both naïve or dpi 2 groups, established marker transcripts for OLs (*Mbp, Plp, Cldn11, Cnp*) or neurons (*NeuN/Rbfox3, Nefh, Nefl* and *Nefm*) showed at least fourfold enrichment or depletion in IP vs. IN samples, respectively (q < 0.05), confirming successful enrichment of OL transcriptome in IP samples.

### qPCR analysis of mRNA expression

RNA was extracted using Trizol or RNeasy Lipid Tissue Mini Kit (Qiagen, Germantown, MD) and cDNA was synthesized following standard methodology. SYBR Green master mix- or Taqman DNA polymerase and ViiA 7 Real-Time PCR System (Applied Biosystems, Grand Island, NY) were used for non-labeled or Taqman primers, respectively (see supplementary Table S2 for primer information). The ΔΔCT method was used for quantification; the normalizing transcript was *Gapdh*. Validation of gene knockouts was performed using custom designed primers to amplify the mRNA regions corresponding to the deleted exon/exons (*Atf4* exons 2–3 or *Chop* exon 3, supplementary Table S2).

### qPCR analysis of genomic DNA

DNA was isolated from frozen spinal cord or liver samples using AllPrep DNA/RNA Mini kit (Qiagen, Germantown, MD. Cat# 80204) according to manufacturer’s recommendations. All primmer sequences are listed in the Supplementary Table S2. To ensure specific amplification, DNA products of a regular PCR were separated using agarose gel electrophoresis and their number and size was determined. PCR (25 cycles) was performed using Promega 5XGreen GoTaq Flexi Buffer (cat# M891A), GoTaq Flexi DNA Polymerase (cat# M829B) and a minicycler PCR instrument (MJ Research INC, Watertown, MA). Then qPCR analyses were conducted for all amplicons using SYBR Green master mix and ViiA 7 Real-Time PCR System (Applied Biosystems, Grand Island, NY). Quality control for those assays included standard curves and melting curves of qPCR products. The ΔΔCT method was used for quantification with a normalizing amplicon from a non-recombining region of the Chop gene (amplicon 3).

### WMS analysis

WMS was evaluated using iron eriochrome cyanine (EC) staining for myelin as described previously^11^. A detailed description is provided in Supplementary Methods.

### Immunostaining

Slides with mounted spinal cord sections were warmed at 37 °C for 10 min, washed 3× with PBS, and blocked in TBS + 0.1% Triton X-100, 0.5% BSA, and 10% normal donkey or goat serum for 1 h at room temperature and then incubated overnight at 4 °C with primary antibodies in blocking buffer (mouse anti-APC, clone CC1 (1:200, Calbiochem, OP80), rabbit anti-OLIG2 (1:200, Millipore, Ab9610), followed by 3× washes and incubation in secondary antibodies at 25 °C for 1 h. Negative controls included appropriate species-specific non-immune Ig subtypes instead of primary antibodies. Cell nuclei were counter-stained with Hoechst-33258. Co-immunostanings for APC/CC1 (1:100, Calbiochem, OP80) and ATF5 (1:100, Novusbio, NBP2-92397) or GPX4 (1:100, Abcam, ab125066) were performed in a similar manner except use of 10% or 5% normal goat serum (in 0.1% Triton X-100, PBS) for blocking or primary antibody incubation, respectively.

### Imaging

The white matter sparing images were captured with Nikon Eclipse Ti inverted microscope equipped using a 4×  objective. The co-immunostaining images were captured with Nikon C2 confocal microscope using a 10× objective for cell counts. Identical acquision parameter were used for corresponding injured and control spinal cords. Digitized images were captured using NIS-Elements software Ar 5.3 followed by conversion to JPEG files.

### OL content

Quantification of fluorescent immunostained images followed previously described methodology using transverse 20 μm-thick sections that were stained with OLIG2 (OL/OPC marker) or CC1 (OL marker) and counterstained with Hoechst-33258^65^.

### Statistical analysis

Repeated measures ANOVA (RM-ANOVA) followed by Bonferroni post hoc tests was used for analyzing locomotor recovery (BMS, ladder test) and histological data (WMS, cell counts). Gait parameters were analyzed using RM-ANOVA/Bonferroni or t-test (two-tailed) as indicated. Transcript expression/genomic amplicon data (qPCR) were analyzed using the non-parametric Mann–Whitney test (*u*-test, two-tailed).

## Supplementary Information

Supplementary Information 1.

## Data Availability

The data that support the findings of this study are available from the corresponding author upon reasonable request.
