# Supplementary material for: Opposite modulation of functional recovery following contusive spinal cord injury in mice with oligodendrocyte-selective deletions of Atf4 and Chop/Ddit3
Source: Sci Rep. 2023 Jun 6;13:9193. doi: 10.1038/s41598-023-36258-2 (PMC10244317; doi:10.1038/s41598-023-36258-2)
Supplement: Supplementary file 1 — Supplementary Information 1. [file 41598_2023_36258_MOESM1_ESM.pdf]

Opposite modulation of functional recovery following contusive spinal cord injury in mice with oligodendrocyte-selective deletions of *Atf4* and *Chop/Ddit3*

Y. Gao et al.

Supplementary Materials:

Supplementary Figures S1-S5

Supplementary Tables S1-S5

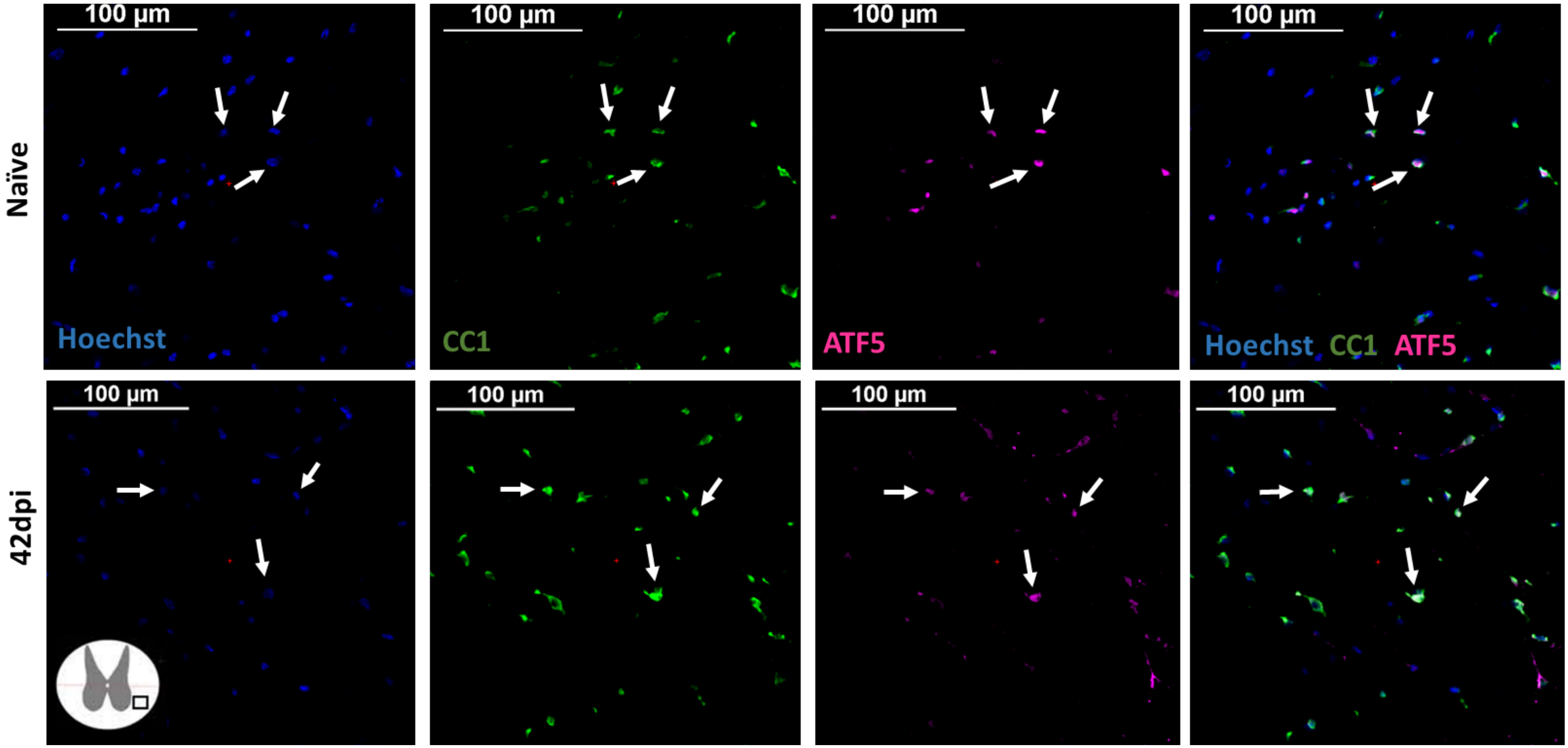

Supplementary Figure S1. Expression of select ISR target gene protein products in OLs from the spinal cord spared white matter on dpi 42: ATF5. Co-immunostainings for the mature OL marker (CC1) and ATF5 were performed using wt (C57Bl6, females) spinal cord tissue collected on dpi 42 as described for Fig. 6. Representative confocal images of ATF5+CC1 co-immunostainings in the spared ventral white matter 1 mm rostral to the injury epicenter; cell nuclei are counterstained with Hoechst-33258. $ATF5^{+}$ OLs are indicated by arrows. Note OL expression of ATF5 both in naïve and SCI mice. Similar staining pattern was observed in 2 additional mice from each group and was also present caudally from the injury epicenter. No immunofluorescence signal was produced when isotype/concentration-matched IgGs was used instead of the primary antibody against ATF5.

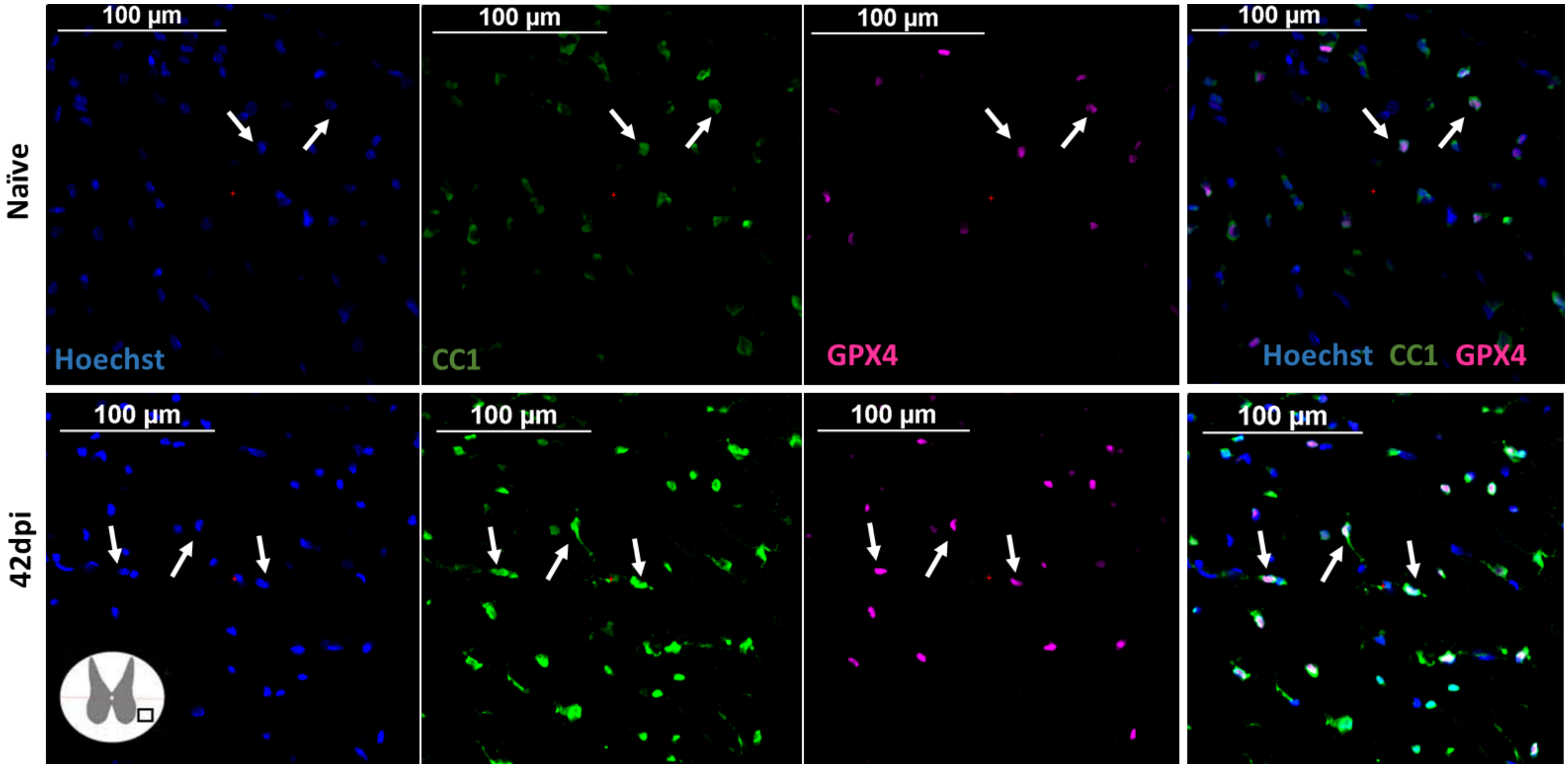

Supplementary Figure S2. Expression of select ISR target gene protein products in OLs from the spinal cord spared white matter on dpi 42: GPX4. Co-immunostainings for the mature OL marker (CC1) and GPX4 were performed using wt (C57Bl6, females) spinal cord tissue collected on dpi 42 as described for Fig. 6. Representative confocal images of GPX4+CC1 co-immunostainings in the spared ventral white matter 1 mm rostral to the injury epicenter; cell nuclei are counterstained with Hoechst-33258. GPX4+ OLs are indicated by arrows. Note OL expression of GPX4 both in naïve and SCI mice. Similar staining pattern was observed in 2 additional mice from each group and was also present caudally from the injury epicenter. No immunofluorescence signal was produced when isotype/concentration-matched IgGs was used instead of the primary antibody against GPX4.

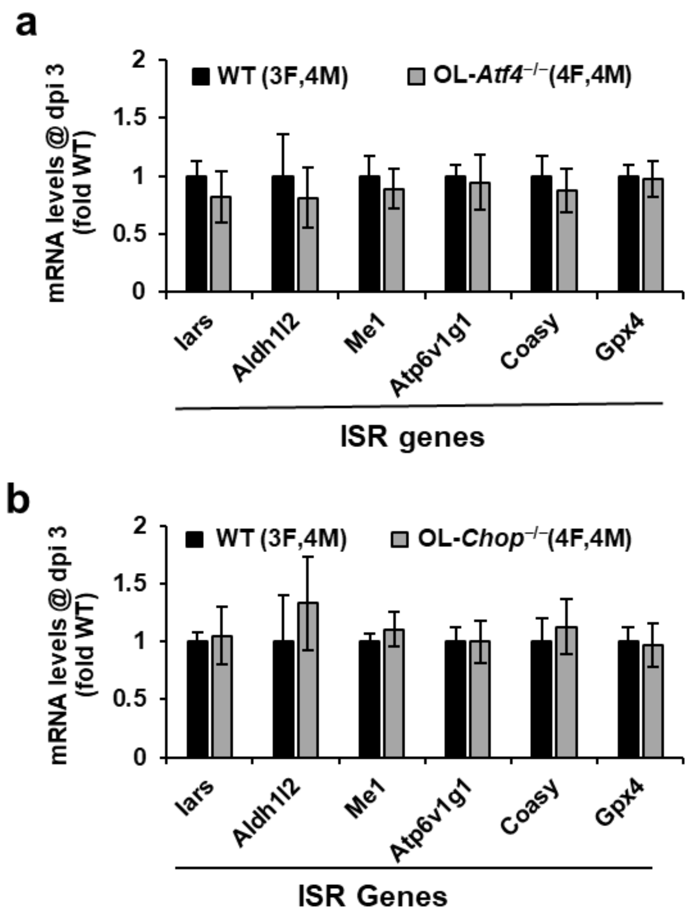

Supplementary Figure S3. Levels of ISR mRNAs in OL-*Atf4*$^{-/-}$ or OL-*Chop*$^{-/-}$ mice on dpi 3. Transcript levels were determined in total spinal cord RNA using qPCR as described for Fig. 2. Expression of select ATF4- and/or CHOP target genes is not significantly affected by OL-*Atf4* or OL-*Chop* KO at dpi 3. Means±SD are shown (wt vs. KO, $p>0.05$, Mann-Whitney *u*-test).

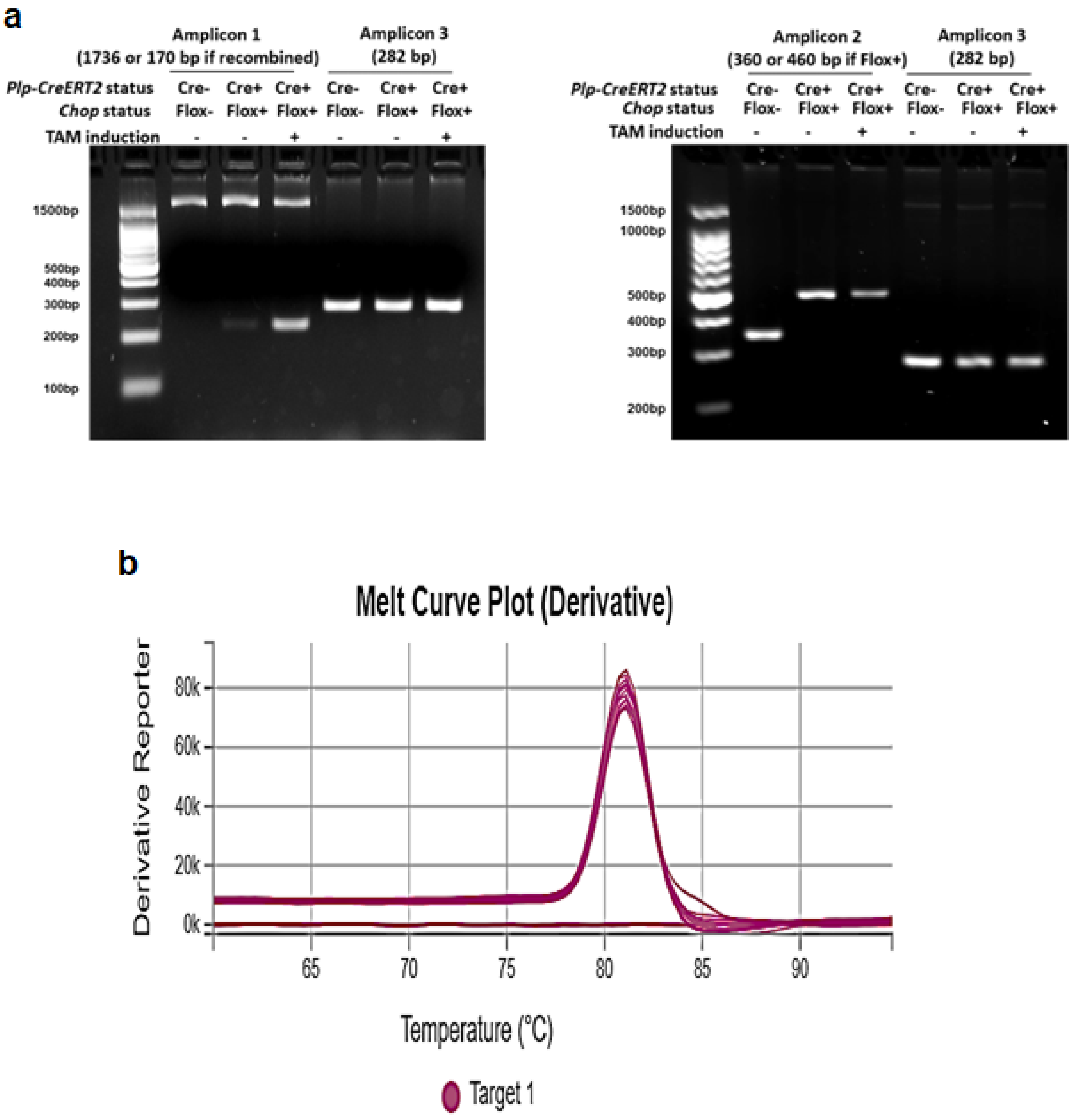

Supplementary Figure S4. Testing genomic DNA amplicons that were used to analyze Cre-mediated recombination of the *Chop* gene. ***(a),*** Agarose gel electrophoresis of the PCR products revealed the expected size and high specificity of amplification. Note that increased size of the amplicon 2 product in vehicle (TAM-) or tamoxifen (TAM+) treated *Plp-CreERT2:Chop*$^{flox/flox}$ mice vs. *Chop*$^{wt/wt}$ (Flox-) mice is caused by the insertion of the loxP in the intron 3 of the *Chop* gene (see Fig. 2e). In addition, a weak amplicon 2 product that is visible in vehicle treated *Plp-CreERT2:Chop*$^{fl/fl}$ mice is likely due to baseline, tamoxifen independent activity of the CreERT2 (see Results for more details). ***(b),*** Melting curve of the qPCR product of the Amplicon 1 indicates presence of a single DNA product. Therefore, under qPCR conditions, the short, 170 bp product that arises after Cre-mediated recombination is favored over the long, 1736 bp product from an unrecombined gene. indicated that each amplicon resulted in a single product.

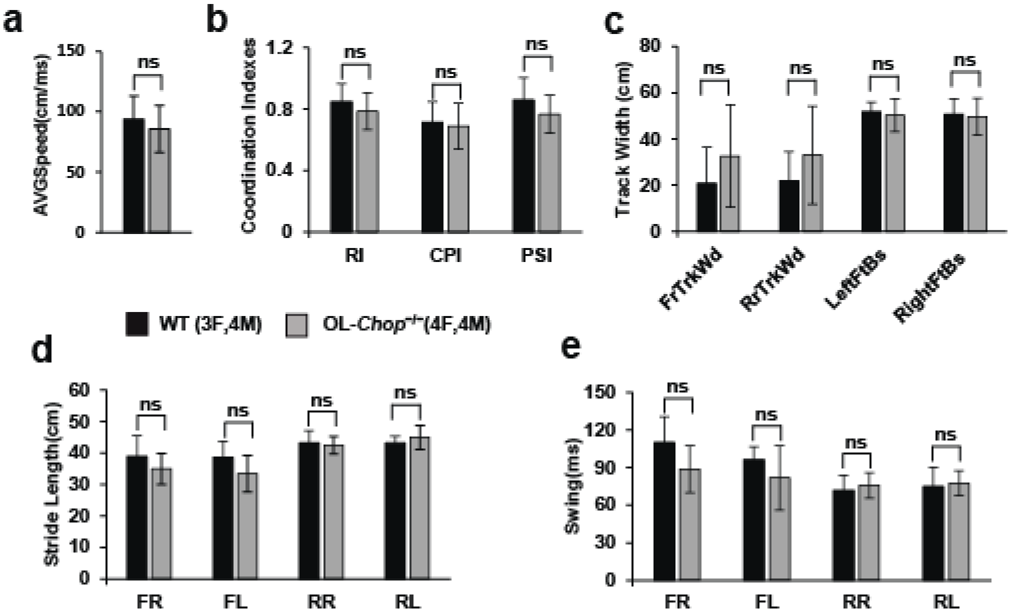

Supplementary Figure S5. Similar gait parameters in wt and OL-*Chop*$^{-/-}$ mice on dpi 42. Analysis of gait was performed as described for Fig. 5. For each gait parameter, mean±SD is shown; ns, $p>0.05$, *t*-test in *a-c* or ANOVA *d*, *e*. No significant effects on any gait parameter were found after SCI in OL-*Chop*$^{-/-}$ mice.

Supplementary Table S1. Parameters of spinal cord contusion for each individual mouse in functional recovery experiments.

Plp-cre-ATF4 mouse chip ID	Genotype	Force (K Dynes)	Displacement (μM)	Velocity (mm/s)	Dwell time (s)
3374	OL-*Atf4*$^{-/-}$	51	546	129	0
5268	OL-*Atf4*$^{-/-}$	53	740	129	0
8596	OL-*Atf4*$^{-/-}$	51	670	127	0
2805	OL-*Atf4*$^{-/-}$	53	529	122	0
3026	OL-*Atf4*$^{-/-}$	51	529	124	0
2307	OL-*Atf4*$^{-/-}$	52	529	115	0
2256	OL-*Atf4*$^{-/-}$	50	582	122	0
5587	WT	53	476	129	0
0580	WT	52	664	117	0
1101	WT	50	493	124	0
2806	WT	52	564	127	0
3039	WT	52	423	129	0
2850	WT	49	493	115	0

No significant differences in force or displacement between OL-*Atf4*$^{-/-}$ and WT mice (two-tail *t*-test, $p>0.05$)

Plp-cre-Chop mouse chip ID	Genotype	Force (K Dynes)	Displacement (μM)	Velocity (mm/s)	Dwell time (s)
3039	OL-*Chop*$^{-/-}$	51	740	132	0
7326	OL-*Chop*$^{-/-}$	50	564	127	0
0080	OL-*Chop*$^{-/-}$	53	529	115	0
3593	OL-*Chop*$^{-/-}$	54	705	129	0
2256	OL-*Chop*$^{-/-}$	50	705	127	0
3598	OL-*Chop*$^{-/-}$	51	440	122	0
3374	OL-*Chop*$^{-/-}$	53	476	127	0
9115	OL-*Chop*$^{-/-}$	51	423	129	0
5587	WT	52	440	122	0
8596	WT	51	529	129	0
0584	WT	52	493	122	0
2806	WT	51	529	117	0
3026	WT	51	705	129	0
1101	WT	50	493	115	0
5268	WT	51	405	132	0
6771	WT	50	670	127	0
2307	WT	52	529	117	0

No significant differences in force or displacement between OL-*Chop*$^{-/-}$ and WT mice (two-tail *t*-test, $p>0.05$)

Supplementary Table S2. List of qPCR primers.

**Gene**	**Assay ID (ThermoFisher Scientific TaqMan Assay)**
Iars	Mm01258263_m1
Aldh1l2	Mm00463499_m1
Me1	Mm07293398_g1
Bcat1	Mm00500289_m1
Coasy	Mm01292513_g1
Chop	Mm01135937_g1
Cnp	Mm01306640_m1
Aspa	Mm00480867_m1
Opalin	Mm00463365_m1
Eno2	Mm01185009_gH
Rbfox3	Mm01248781_m1
Gfap	Mm01253033_m1
Serpina3n	Mm00776439_m1
Atp6v1g1	Mm05695784_g1
**Gene/mRNA**	**Primer sequences used for SybrGreen reactions**
Atf4 mRNA	Forward 5’GGTTCTCCAGCGACAAGG3’<br>Reverse 5’GCATCGAAGTCAAACTCTTTCAG3’
Chop gene<br>*Amplicon 1*	Forward 5'CCCAACTCTCAGTCCACACC 3'<br>Reverse 5'GGAGAGGCATACAAACCCCC 3'
Chop<br>*gene/*mRNA<br>*Amplicon 2*	Forward 5’GCACCTATATCTCATCCCCAG3’<br>Reverse 5’TGCGTGTGACCTCTGTTG3’
Chop gene<br>*Amplicon 3*	Forward 5’ CCAGAATTCCAGGATGCCCA3’<br>Reverse 5’ GGTGGCCAAGATTACCTGCT3’

Supplementary Table S3. Results of the Repeated Measures ANOVA main effects (Factors 1 (F1) and 2 (F2) and their interaction (F1xF2)) are shown in the top panel for each of the BMS, BMS subscore and horizontal ladder assessments for Atf4-/- and Chip-/-. Significant *post hoc* t-test comparisons (Bonferroni) are listed in the lower panel. D=Day; W=Week

Atf4-/-		Chop-/-	
**BMS**		**BMS**	
F1: Genotype	$F_{(1,44)}=3.2$, $p>0.05$	F1: Genotype	$F_{(1,76)}=3.7$, $p=0.058$
F2: Time after Injury	$F_{(6,27)}=12.1$, $p<0.001$	F2: Time after Injury	$F_{(6,31)}=39.6$, $p<0.001$
F1xF2	$F_{(6,27)}=1.8$, $p>0.05$	F1xF2	$F_{(6,31)}=0.37$, $p>0.05$
**BMS Subscore**		**BMS Subscore**	
F1: Genotype	$F_{(1,10)}=5.9$, $p<0.05$	F1: Genotype	$F_{(1,48)}=0.01$, $p>0.05$
F2: Time after Injury	$F_{(5,10)}=2.7$, $p>0.05$	F2: Time after Injury	$F_{(5,19)}=0.74$, $p>0.05$
F1xF2	$F_{(5,10)}=1.1$, $p>0.05$	F1xF2	$F_{(5,19)}=0.90$, $p>0.05$
**LADDER**		**LADDER**	
F1: Genotype	$F_{(1,27)}=22.5$, $p<.001$	F1: Genotype	$F_{(1,30)}=10.1$, $p<0.005$
F2: Time after Injury	$F_{(3,16)}=86.3$, $p<0.001$	F2: Time after Injury	$F_{(3,32)}=82.8$, $p<0.001$
F1xF2	$F_{(3,16)}=9.7$, $p<0.001$	F1xF2	$F_{(3,32)}=6.3$, $p<0.005$

Atf4-/-		Chop-/-	
**BMS**		**BMS**	
F1: Genotype	N/A	F1: Genotype	N/A
F2: Time after Injury		F2: Time after Injury	
WT: D4 < D7, p<0.01		WT: D3 < W1-W6, p<0.001	
Atf4-/-: D4 < D7, p<0.01		WT: W1 < W3, p<0.05	
Atf4-/-: D4 < D14-D42, p<0.001		WT: W1 < W5, p<0.05	
		Chop-/-: D3 < W1-W6, p<0.001	
**BMS Subscore**		**BMS Subscore**	
F1: Genotype	D14: Atf4-/- > WT, p<0.05	F1: Genotype	None
F2: Time after Injury	N/A	Time After Injury	N/A
**LADDER**		**LADDER**	
F1: Genotype		F1: Genotype	
W2: Atf4-/- > WT, p<0.05		W4: Chop-/- < WT, p<0.01	

W4: Atf4-/- > WT, p<0.005<br>W6: Atf4-/- > WT, p<0.05<br>F2: Time after Injury<br>WT: BL < W2, p<0.005<br>WT: BL < W6, p<0.005	W6: Chop-/- < WT, p<0.01<br>F2: Time after Injury<br>WT: BL < W2, p=0.001<br>WT: BL < W4, p<0.001<br>WT: BL < W6, p<0.001
Atf4-/-: BL < W2, p<0.001<br>Atf4-/-: BL < W4, p<0.001<br>Atf4-/-: BL < W6, p<0.001<br>Atf4-/-: W2 < W4, p<0.05<br>Atf4-/-: W2 < W6, p<0.01	Chop-/-: BL < W2, p<0.01<br>Chop-/-: BL < W4, p<0.005<br>Chop-/-: BL < W6, p<0.001

Supplementary Table S4. Results of the Repeated Measures ANOVA main effects (Factors 1 (F1) and 2 (F2) and their interaction (F1xF2)) are shown in the top panel for week 6 post-injury Stride Length, Swing and Base of Support kinematic treadscan assessments of the forelimb (F-L, top) and hindlimb (H-L, middle) for Atf4-/-. Significant post hoc t-test comparisons (Bonferroni) are listed to the right. Independent t-tests were performed comparing the Genotypes' Coordination, Track Width and Walking Speed in the lower panel. D=Day; W=Week

ANOVA			*post hoc t-test*	
**STRIDE LENGTH**	F-L		F1: Genotype	
F1: Genotype		$F_{(1,22)}$=35.1, p<.001	Right Side: WT > Atf4-/-, p<.005	
F2: Side		$F_{(1,22)}$=0.02, p>0.05	Left Side: WT > Atf4-/-, p=.001	
F1xF2		$F_{(1,22)}$=0.53, p>0.05	F2: Side	N/A
**SWING**	F-L		F1: Genotype	
F1: Genotype		$F_{(1,22)}$=31.5, p<.001	Right Side: WT > Atf4-/-, p<.005	
F2: Side		$F_{(1,22)}$=0.02, p>0.05	Left Side: WT > Atf4-/-, p<.005	
F1xF2		$F_{(1,22)}$=0.012, p>0.05	F2: Side	N/A
**BASE OF SUPPORT**				
F1: Genotype		$F_{(1,22)}$=1.0, p>0.05	F1: Genotype	N/A
F2: Side		$F_{(1,22)}$=0.01, p>0.05	F2: Side	N/A
F1xF2		$F_{(1,22)}$=0.00, p>0.05		
**STRIDE LENGTH**	H-L			
F1: Genotype		$F_{(1,22)}$=7.4, p<0.05	F1: Genotype	
F2: Side		$F_{(1,22)}$=0.00, p>0.05	Right Side: WT > Atf4-/-, p=.056	
F1xF2		$F_{(1,22)}$=0.10, p>0.05		
**SWING**	H-L			
F1: Genotype		$F_{(1,21)}$=3.7, p>0.05	F1: Genotype	N/A
F2: Side		$F_{(1,21)}$=1.3, p>0.05	F2: Side	N/A
F1xF2		$F_{(1,21)}$=0.01, p>0.05		

INDEPENDENT T-TEST			
**COORDINATION**		**TRACK WIDTH**	
Regularity Index (RI)	$t_{(8)}$=1.3, p>0.05*	Front Track Width	$t_{(11)}$=0.95, p>0.05
Coord. Pattern Index (CPI)	$t_{(11)}$=1.3, p>0.05	Rear Track Width	$t_{(11)}$=3.0, p<0.05
Plantar Stepping Index (PSI)	$t_{(11)}$=3.3, p>0.01	Avg Speed	$t_{(11)}$=2.5, p<0.05

*corrected for unequal variance

Supplementary Table S5. Results of statistical analysis of WMS, CC1+, and OLIG2+ cell count data. RM-ANOVA F values and Bonferroni post-hoc tests are presented.

**wt vs. OL-*Atf4*$^{-/-}$**	**wt vs. OL-*Chop*$^{-/-}$**
WMS<br>F1: genotype $F(1,9)=1.79$, $p=0.23$<br>F2: distance to epi. $F(2,18)=120.64$, $p<0.001$<br>F1xF2 $F(2,18)=0.01$, $p=0.98$	WMS<br>F1: genotype $F(1,12)=0.635$, $p=0.44$<br>F2: distance to epi. $F(2,24)=94.18$, $p<0.001$<br>F1xF2 $F(2,24)=0.57$, $p=0.57$
OL content (CC1+)<br>F1: genotype $F(1,10)=68.58$, $p<0.001$<br>F2: distance to epi. $F(2,20)=219.40$, $p<0.001$<br>F1xF2 $F(2,20)=18.38$, $p<0.001$	OL content (CC1+)<br>F1: genotype $F(1,12)=7.62$, $p<0.05$<br>F2: distance to epi. $F(2,24)=23.73$, $p<0.001$<br>F1xF2 $F(2,24)=0.23$, $p=0.79$
OL linage cell content (OLIG2+)<br>F1: genotype $F(1,10)=32.54$, $p<0.001$<br>F2: distance to epi. $F(2,20)=121.82$, $p<0.001$<br>F1xF2 $F(2,20)=18.40$, $p<0.001$	OL linage cell content (OLIG2+)<br>F1: genotype $F(1,12)=11.29$, $p<0.01$<br>F2: distance to epi. $F(2,24)=24.36$, $p<0.001$<br>F1xF2 $F(2,24)=0.05$, $p=0.95$
WMS, post-hoc<br>F1 N/A<br>F2 -1 mm > Epi< +1 mm in wt or OL-*Atf4*$^{-/-}$:	WMS, post-hoc<br>F1 N/A<br>F2 -1 mm > Epi< +1 mm in wt or OL-*Chop*$^{-/-}$
OL content (CC1+), post-hoc<br>F1 wt > OL-*Atf4*$^{-/-}$ @ +1 mm<br>F2 -1 mm > Epi< +1 mm in wt or OL-*Atf4*$^{-/-}$<br>-1 mm<+1 mm in wt, but not OL-*Atf4*$^{-/-}$	OL content (CC1+), post-hoc<br>F1 no significant differences<br>F2 -1 mm > Epi< +1 mm in wt or OL-*Chop*$^{-/-}$
OL linage cell content (OLIG2+)<br>F1 wt > OL-*Atf4*$^{-/-}$ @ +1 mm<br>F2 -1 mm > Epi< +1 mm in wt or OL-*Atf4*$^{-/-}$<br>-1 mm<+1 mm in wt, but not OL-*Atf4*$^{-/-}$	OL linage cell content (OLIG2+)<br>F1 no significant differences<br>F2 -1 mm > Epi< +1 mm in wt or OL-*Chop*$^{-/-}$
